# The Effect of Medication Self-Management During Hospitalisation (SelfMED) on Medication Adherence Post-Discharge in Oncology Patients with Polypharmacy: A Prospective, Exploratory, Single-Centre, Non-Randomised Pre–Post Intervention Study with Sequential Control and Intervention Cohorts

**DOI:** 10.3390/healthcare14203436

**Published:** 2026-10-14

**Authors:** Laura Mortelmans, Alisa Schenkel, Eva Goossens, Tinne Dilles

**Affiliations:** 1 Centre for Research and Innovation in Care (CRIC), Department of Nursing and Midwifery Sciences, Faculty of Medicine and Health Sciences, University of Antwerp, 2610 Antwerp, Belgiumeva.goossens@uantwerpen.be (E.G.); tinne.dilles@uantwerpen.be (T.D.); 2 Research Foundation Flanders (FWO), 1000 Brussels, Belgium; 3 Nurse and Pharmaceutical Care Consortium, NuPhaC, 2610 Antwerp, Belgium; 4 Oncology Department, Jessa Ziekenhuis, 3500 Hasselt, Belgium; 5 Department of Patient Care, Antwerp University Hospital (UZA), 2610 Antwerp, Belgium

**Keywords:** medication adherence, self-management, polypharmacy, hospitalisation, oncology, patient empowerment

## Abstract

**Objectives**: To evaluate the effect of in-hospital medication self-management (SelfMED) on medication adherence two months post-discharge in polymedicated oncology patients. **Methods**: A prospective, exploratory, single-centre non-randomised pre–post intervention study with sequential control and intervention cohorts was conducted. During a five-month control phase, patients received usual care, in which medication was managed by healthcare providers. In the subsequent five-month intervention phase, patients self-managed their medication during hospitalisation using the SelfMED procedure. SelfMED consists of: (1) an eligibility assessment for self-management, (2) a monitoring tool to follow up medication intake and detect problems and (3) a support tool for healthcare providers to assist patients with problems. Medication adherence was assessed two months post-discharge using pill counts and self-report. Secondary outcomes included self-management problems, medication knowledge, patient and healthcare provider satisfaction, workload and healthcare service utilisation. **Results**: Sixty-four patients completed the study (control: n = 34; intervention: n = 30). Two months post-discharge, patients in the intervention group were, on average, adherent to 74.5% of their prescribed medications, versus 59.1% in the control group (difference 15.4 percentage points; 95% CI −0.8 to 31.7). This difference was not statistically significant (*p* = 0.062). Patients in the intervention group reported fewer medication self-management problems in their diaries (3.3% vs. 26.5%, *p* = 0.015) and more often felt adequately prepared for medication self-management at home (93.1% vs. 71.4%, *p* = 0.041). No differences were observed in medication knowledge, healthcare service utilisation, or workload. **Conclusions**: SelfMED did not significantly improve medication adherence after discharge but may support patients’ preparedness for medication self-management at home. Given the small achieved sample size, these findings should be regarded as exploratory. Studies with larger samples and more diverse populations are needed to confirm these findings.

## 1. Introduction

Medication adherence, defined as “the process by which patients take their medicines as prescribed” [1], remains a persistent challenge in clinical practice, with significant implications for both health outcomes and healthcare costs [2,3]. The World Health Organization estimates that approximately half of patients with chronic conditions fail to adhere to their prescribed medication regimens [4]. More recent data in individuals with multimorbidity indicate non-adherence rates ranging from 7% to 83.5%, reflecting considerable variability across conditions, treatments and measurement methods [5]. Similar variability has been observed among patients receiving self-administered cancer treatments. For example, a systematic review of oral anticancer therapies reported adherence rates ranging from 46% to 100% [6].

One factor that may contribute to non-adherence is polypharmacy. Polypharmacy has been associated with non-adherence, as it increases regimen complexity and is particularly prevalent among patients with chronic illnesses [7]. Polypharmacy is common among patients with cancer [8,9,10], owing to the need for supportive therapies—such as antiemetics and analgesics—to manage the side effects of anticancer agents [9,11]. The prevalence of polypharmacy among patients with cancer has been reported to range from approximately 20% to 80% [12]. Poor adherence to supportive medications may exacerbate side effects, potentially resulting in treatment interruptions or premature discontinuation of anticancer therapy. Moreover, medication regimens are frequently modified during hospital admissions, for example when supportive medicines are initiated or adjusted, making the transition from hospital to home a particularly vulnerable period for medication adherence.

Medication self-management (MSM) involves a set of interrelated knowledge, skills, motivation, and behaviours required to follow prescribed regimens consistently. Bailey’s model describes MSM as a six-step process: obtaining prescriptions, understanding medicines, organising and scheduling intake, administering doses, monitoring therapeutic and adverse effects, and sustaining safe use [13]. Although MSM and medication adherence are distinct concepts, they are interrelated. Difficulties in the MSM process—such as forgetting doses, misunderstanding instructions, or problems handling medication—can be related to non-adherence [14]. Therefore, interventions designed to strengthen MSM are expected to improve medication adherence.

Hospitalisation often limits opportunities for MSM because healthcare providers typically manage patients’ medicines. However, patients are expected to resume self-management responsibilities after discharge [15]. As a result, hospital stays may disrupt the continuity of MSM practices and increase the risk of drug-related problems (DRPs) at home, with the reported prevalence of post-discharge DRPs ranging from 14% to 49% [16]. Studies indicate that 24% to 70% of patients are non-adherent to their medication after discharge [17,18].

To address this challenge, in-hospital MSM programmes have been developed to allow patients to self-store, -prepare and -administer medication under the supervision of healthcare providers during their hospital stay. Such programmes enable healthcare providers to monitor and support patients, and to identify and address medication-related problems together with the patient before discharge, with the aim of strengthening patients’ MSM competencies, reducing DRPs and promoting safe and effective medication use at home [19,20,21]. Evidence suggests that in-hospital MSM may reduce medication errors [19,22], improve medication adherence [19,20], and enhance medication knowledge [19,23].

Despite its potential benefits, in-hospital MSM is not standard practice in Belgium. A cross-sectional study in 12 Flemish hospitals found that only 22% of 1,269 patients self-managed at least one medication during hospitalisation. Where MSM was permitted, implementation rarely followed a structured, evidence-based approach: fewer than 20% of wards had a procedure for MSM and fewer than 10% used a screening tool to assess patients’ eligibility for MSM [24].

To address these gaps, the SelfMED intervention was developed [25]. This multi-component intervention includes a procedure outlining the MSM process and associated responsibilities, an eligibility assessment, a monitoring system and supportive measures for healthcare providers (see Methods for a detailed description). Pilot-based results suggest that the SelfMED-intervention facilitates MSM and can be implemented safely [26]. However, although its feasibility and safety have been demonstrated, there is currently no evidence regarding SelfMED’s effectiveness on post-discharge outcomes, including medication non-adherence.

This study primarily aimed to assess the impact of the SelfMED intervention on medication adherence in patients with polypharmacy two months after hospital discharge, compared with standard care (i.e., medication management by healthcare providers). Secondary aims were to evaluate the effects of SelfMED on patient-level outcomes (i.e., self-management, medication knowledge, and satisfaction), healthcare provider-level outcomes (i.e., staff satisfaction and workload), and healthcare system-level outcomes (i.e., healthcare service utilisation). The study hypothesis was that SelfMED would lead to improved medication adherence at two months post-discharge, compared with standard care.

## 2. Materials and Methods

### 2.1. Design

A prospective, exploratory, single-centre, non-randomised pre–post intervention study was conducted, consisting of a five-month control phase followed by a five-month intervention phase (sequential cohorts). During the control phase, standard care, defined as medication management by healthcare providers, was evaluated (see Section 2.4). Subsequently, several training sessions were held to prepare healthcare providers for the implementation of the SelfMED intervention. During the intervention phase, eligible patients were allowed to self-manage their medications during hospitalisation, with close monitoring and support from the healthcare team (see Section 2.6). Patients in both study phases were followed up for two months post-discharge to assess medication adherence.

Both cohorts were recruited from the same oncology ward using identical eligibility criteria (see Section 2.3). Participants were assigned to the control or intervention group according to the calendar period during which they were admitted to the ward (control period: February to June 2024; intervention period: October 2024 to February 2025). Random allocation was not used, as a pre–post design was chosen to minimise the risk of contamination bias between study groups. Given the achieved sample size, this study should be considered an exploratory evaluation of the SelfMED intervention rather than a confirmatory effectiveness trial.

### 2.2. Study Setting and Sampling

The study was conducted on an oncology ward of a general hospital in Belgium (Flanders). All patients admitted to the oncology ward were screened for eligibility. Those who met the inclusion criteria (see Section 2.3) and provided informed consent were enrolled consecutively.

The sample size calculation was based on the study’s primary outcome: medication adherence two months post-discharge, measured via pill counts. The intervention was considered successful if medication adherence in the intervention group significantly exceeded that in the control group. To estimate the effect size, we referred to a meta-analysis of 771 intervention studies on medication adherence, which reported a standardised mean difference of 0.29 between intervention and control groups [27].

Using G*Power version 3.1.9.7, a sample of 376 participants (188 per group) was required to detect an effect size of 0.29 with 80% power at a two-sided significance level of 0.05. Accounting for an expected attrition rate of 20%, based on prior research on medication adherence in a polypharmacy population [28], the final target sample size was set at 235 patients per group.

### 2.3. Inclusion and/or Exclusion Criteria

Dutch-speaking patients aged 18 years or older were eligible if they were taking five or more chronic medications at hospital admission, of which at least three were administered orally. They were expected to be hospitalised for a minimum of three days. Medicines were considered chronic if the anticipated treatment period was three months or longer.

Patients were excluded if they were returning to a nursing home, had a neuropsychiatric diagnosis of dementia or a Mini Mental State Examination score below 24/30, had a terminal illness with a life expectancy of less than three months, or were unable to provide informed consent. Additionally, only patients who met the criteria for MSM according to the SelfMED assessment tool were included.

### 2.4. Control

Participants included during the control period (February–June 2024) received usual care, in which medication management during the hospital stay was provided exclusively by healthcare providers (i.e., storage, preparation, and administration).

### 2.5. Implementation of the Intervention

Four training sessions were organised between August and September 2024 to inform healthcare professionals about the study objectives and the intervention protocol. During these sessions, healthcare providers were instructed on how to complete the SelfMED assessment and were informed about the prerequisites and practical organisation of in-hospital MSM by patients, as well as the use of the SelfMED monitoring and support system. All full-time nurses attended the training at least once. In addition, healthcare professionals were provided with a video recording of the session and a written standardised operating procedure. A trained research assistant (A.S.) was available on the hospital ward to support the implementation of the intervention.

### 2.6. Intervention

**Figure 1:**
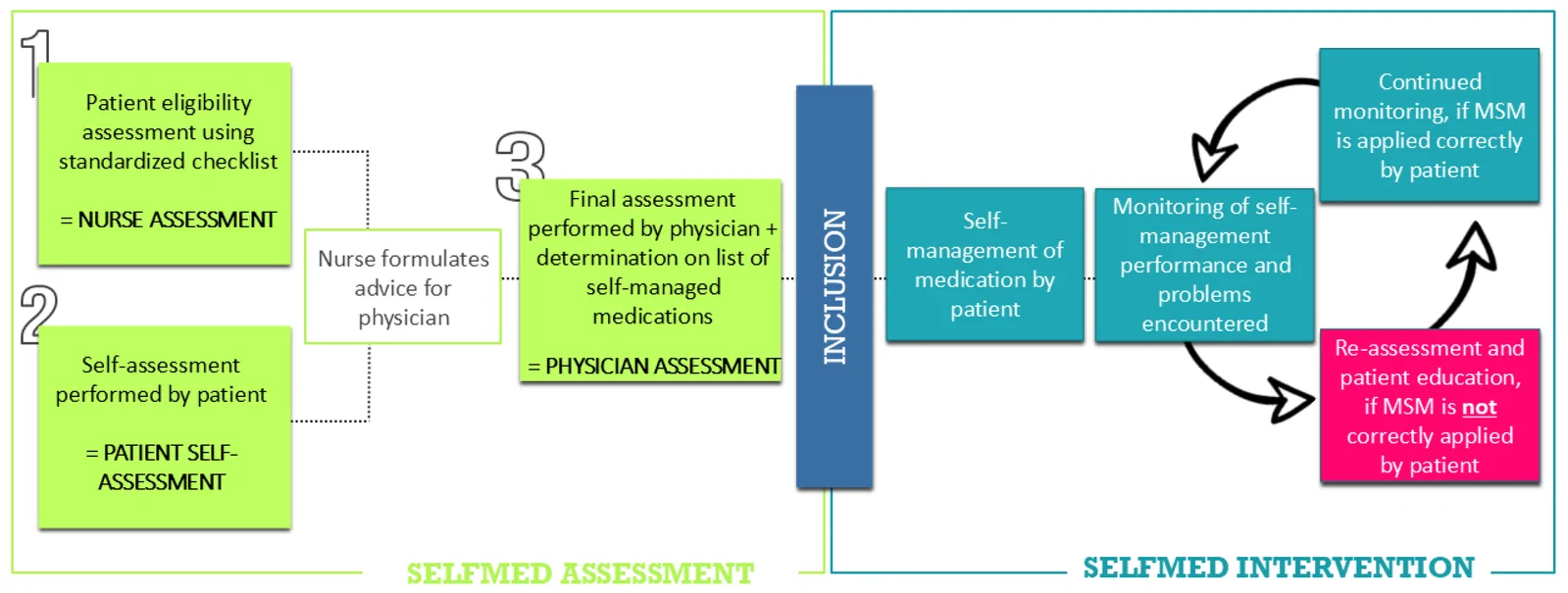
Overview of SelfMED intervention components.

For participants enrolled during the intervention period (October 2024–February 2025), an in-hospital MSM programme (i.e., SelfMED) was implemented with close monitoring of patients’ medication-taking behaviour. SelfMED is an evidence-based, multi-component intervention (Figure 1) [25]:

1. **SelfMED assessment**: A validated stepwise assessment used to evaluate patients’ eligibility for MSM during hospitalisation, conducted by (1) a nurse, (2) the patient and (3) the treating physician. Upon admission, the nurse completed a 10-item checklist assessing factors such as the patient’s cognitive and physical capabilities, prior experience with MSM at home, and the likelihood of returning home after discharge. Subsequently, the patient completed a self-assessment exploring how they currently managed their medication at home, willingness to continue MSM during hospitalisation, and anticipated need for support. Based on both assessments, the nurse formulated a recommendation for the physician. As the physician retains legal responsibility during hospitalisation, the final decision to approve or decline MSM rested with the treating physician, who considered the nurse’s recommendation alongside both assessments.
2. **Initiation of MSM:** For patients deemed eligible for MSM, several practical arrangements were required, including documentation of the self-managed medication in the patient’s medical file, provision of a personalised medication list, delivery of the correct medications to the patient’s room, counselling on self-managed medication and the use of the SelfMED monitoring system.
3. **SelfMED monitor:** A monitoring system enabling healthcare providers to follow up self-managed medication intake and identify potential medication errors or other DRPs. Patients were instructed to record their daily medication intake during their hospital stay, using a medication list similar to that provided at discharge. Patients were allowed to use any aids they used at home during hospitalisation. During medication rounds, nurses reviewed the patient’s entries on the medication list and compared them with the prescribed medication regimen to identify discrepancies. If inconsistencies or concerns regarding adherence were noted, a pill count was performed by a nurse to verify intake. Any issues or difficulties encountered by patients while managing their medicines were discussed directly with them.
4. **SelfMED support:** A guidance document developed to assist healthcare providers in supporting MSM [29]. The guidance document outlines potential problems related to MSM and provides evidence- and expert consensus-based recommendations for addressing these issues. The recommendations were also made available to patients in lay language through an information leaflet.

Further details of the intervention are described in the published protocol [30].

Standard pharmaceutical care was provided in both study groups and included medication reconciliation, electronic prescribing by physicians according to the hospital formulary, medication dispensing by the hospital pharmacy, provision of a medication list at discharge and routine discharge education.

### 2.7. Blinding and Randomisation

Blinding of healthcare providers or participants was not possible, as both groups would have been aware of changes in the method of medication management (i.e., whether it was provided by healthcare providers or by patients themselves). In addition, random allocation of patients to the intervention or control group was not used to avoid contamination bias (i.e., the potential influence of the intervention on the usual care provided to the control group). Participants were assigned to the control or intervention group according to a pre–post design, based on the calendar period in which they were admitted (control: February–June 2024; intervention: October 2024–February 2025). Therefore, the research team members conducting the two-month home visits and pill counts could not be blinded to participants’ group allocation.

### 2.8. Fidelity of the Intervention

Fidelity to the intervention components was qualitatively assessed for each participant upon study completion, based on the documentation provided by healthcare professionals and participants. This assessment entailed verifying whether the SelfMED assessment had been completed by the nurse, patient and physician, determining whether daily monitoring had been conducted in accordance with the SelfMED monitoring form and confirming that appropriate measures had been taken in response to any identified issues in MSM, as documented in the follow up form.

### 2.9. Data Collection and Instruments

Data collection followed a structured, multi-step process. One of the authors (A.S.) screened patients for eligibility during both the control and intervention periods based on the inclusion and exclusion criteria. Eligible patients were invited to participate. After patients had provided informed consent, the SelfMED assessment was completed. Only those who met the eligibility criteria according to the SelfMED assessment were enrolled. Reasons for ineligibility and non-response were recorded.

Upon admission, baseline data on medication adherence, self-management, and medication knowledge were collected. In addition, several potential covariates were collected including age, gender, highest level of education, hospitalisation type and reason, and the number and type of prescribed medicines upon admission. Medication intake of participants in the intervention group was monitored using the SelfMED monitoring tool, which comprised the patient’s medication intake log and the nurse’s pill counts. Data on medicines prescribed at discharge were obtained from a copy of the discharge medication list.

Two months post-discharge, medication adherence, MSM, medication knowledge and healthcare service utilisation were assessed. For this purpose, a home visit was conducted by L.M. or A.S.

#### 2.9.1. Primary Outcome

The primary outcome was medication adherence two months post-discharge, measured using pill counts based of retained medication packages. Medication adherence was defined as “the process by which patients take their medicines as prescribed”, and is typically conceptualised across three phases: initiation, implementation, and discontinuation of therapy [1]. *Initiation* refers to the point at which the patient takes the first dose of a prescribed medication. *Implementation* denotes the extent to which a patient’s actual dosing corresponds to the prescribed dosing regimen, from initiation until the last dose is taken. *Discontinuation* refers to the point at which the patient stops taking the prescribed medication regimen for whatever reason. This study primarily focused on the implementation phase.

The primary outcome was operationalised as the percentage of prescribed chronic medications to which the participant was classified as adherent (i.e., 80–120% of prescribed doses taken; see Section 2.10). This outcome therefore reflects the proportion of medications taken adequately, rather than the overall percentage of doses taken.

To ensure consistency of adherence assessments, all available medication packages were systematically reconciled with the participant’s medication list following each home visit, and any medication changes during follow-up were verified using the available medication lists. Adherence calculations were subsequently performed according to predefined rules.

Medications prescribed on an as-needed basis were excluded from adherence calculations. For newly initiated medications expected to be taken for at least three months, adherence was assessed from the date of prescription onwards. Medication changes occurring during follow-up were considered during pill-count assessment: for medications that were started, stopped or changed in dose, the expected number of doses was calculated separately for the period during which each regimen applied. For participants who were readmitted to hospital during follow-up, inpatient treatment days were excluded from adherence calculations. Missing medication packages were counted as doses not taken.

#### 2.9.2. Secondary Outcomes

In addition to pill counts, **patient-reported medication adherence** was assessed, as evidence suggests that combining multiple methods yields the most accurate measurement of medication adherence [31]. The Probabilistic Medication Adherence Scale (ProMAS) [32] was completed at baseline (i.e., hospital admission) and two months post-discharge. Additionally, participants completed a structured medication diary in which they recorded any problems with medication use [33].

**Problems related to MSM** were evaluated at baseline and two months post-discharge using a self-report questionnaire. Items were developed according to the six phases of Bailey’s MSM model [13] and were based on previously identified MSM problems [29]. (The questionnaire is provided in the published study protocol [30]).

**Medication knowledge** was assessed at baseline and two months post-discharge. Consistent with previous studies that assessed medication knowledge using a randomly selected subset of medications [34, 35,36,37], four medicines were randomly selected from each participant’s medication list. For each medication, patients were asked to identify the medication name, indication, dosage, administration route and timing of administration. Each element was scored as correct (1) or incorrect (0). A total medication knowledge score was calculated as the sum of all responses.

**Patient satisfaction** with pharmaceutical care during hospitalisation was measured at discharge using a visual analogue scale (0–10).

**Healthcare providers’ satisfaction with pharmaceutical care**: Nurses and physicians completed a survey at the end of the study comprising both visual analogue scales and open-ended questions. The open-ended questions explored perceived advantages and disadvantages of usual care compared with MSM.

**Healthcare providers’ perceived workload**: Nurses and physicians rated the feasibility and perceived time investment of both usual care and MSM using 10-point numerical rating scales.

**Healthcare service utilisation**: Medication-related advice-seeking behaviour and healthcare service utilisation in the two months after discharge, including the number of hospital readmissions, nurse consultations, and consultations with general practitioners, were assessed using a self-report questionnaire.

All outcomes were assessed in both the intervention and control groups, with the exception of healthcare providers satisfaction and workload.

### 2.10. Data Analysis

Data were analysed using IBM SPSS Statistics, version 31. The normality of the data was assessed using Z-scores for skewness and kurtosis, with values between −3.29 and +3.29. Frequency distributions were used to describe categorical variables, whereas means and standard deviations (parametric data) or median and range (non-parametric data) were reported for continuous variables. Socio-demographic characteristics were compared between intervention and control groups using Independent *T*-tests or Mann–Whitney U tests, Fisher’s Exact test and Pearson Chi-square tests, as appropriate. Two-sided *p*-values < 0.05 were considered statistically significant. Missing data were handled using complete-case analysis (listwise deletion).

Primary and secondary outcomes were analysed using complete-case analysis. Participants lost to follow-up were excluded from the analysis, as no outcome data were available at the two-month follow-up. Therefore, a conventional intention-to-treat analysis was not performed. To assess the robustness of the primary outcome to attrition, post hoc sensitivity analyses were performed in which missing primary outcome data for participants lost to follow-up were imputed under three scenarios: (i) worst case, in which all dropouts in both groups were assigned 0% adherence; (ii) extreme scenario unfavourable to the intervention, in which dropouts in the intervention group were assigned 0% and dropouts in the control group 100%; and (iii) cross-over scenario, in which dropouts were assigned the observed mean of the other group.

The primary outcome, medication adherence measured using pill counts, was calculated as follows: (the assumed number of pills taken based on retained medication packages/the number of pills supposed to be taken according to the prescription) × 100. Adherence rates were calculated per medicine. A threshold of 80–120% pills taken was used to distinguish between adherence and non-adherence [38,39]. Adherence values below 80% or above 120% were classified as non-adherent, reflecting potential medication underuse or overuse. Subsequently, the average percentage of medicines to which patients were adherent was calculated. Differences in medication adherence between the intervention and control groups were tested using an Independent *T*-test. The Anatomical Therapeutic Chemical (ATC) classification was used to categorise medicines based on the organ system they act upon [40]. Differences in medication adherence across ATC categories (adherent vs. non-adherent) were examined using the Chi-squared test. To account for multiple testing, a Bonferroni correction was applied to the between-group comparisons per ATC category.

Secondary outcomes included continuous variables, i.e., the number of problems reported in the medication diary, the sum score on the ProMAS survey, the sum score for medication knowledge, the number of MSM problems, satisfaction and workload ratings on a 10-point scale, and the number of readmissions, consultations, and nurse visits. Depending on data normality, differences between intervention and control groups were evaluated using either Independent *T*-tests or Mann–Whitney U tests. Within-group comparisons between baseline and two months post-discharge were conducted using Paired *T*-tests or Wilcoxon Signed Rank tests. In addition to *p*-values, effect estimates with corresponding 95% confidence intervals were reported, including mean differences for normally distributed continuous outcomes, Hodges–Lehmann median differences for non-normally distributed outcomes, and odds ratios for dichotomous outcomes.

Univariable linear regression analyses were used to identify factors potentially associated with patients’ medication adherence. All socio-demographic characteristics, study group (intervention vs. control), number of self-management problems, and medication knowledge were included as independent variables. Categorical variables were coded as dummy variables where appropriate. Although no significant associations were identified in the univariable analyses, a multivariable linear regression model was constructed, with medication adherence at two months post-discharge as the dependent variable. Study group, number of prescribed medicines at admission, age, gender and educational level were entered as independent variables based on clinical relevance.

## 3. Results

### 3.1. Participant Flow and Sample Characteristics

**Figure 2:**
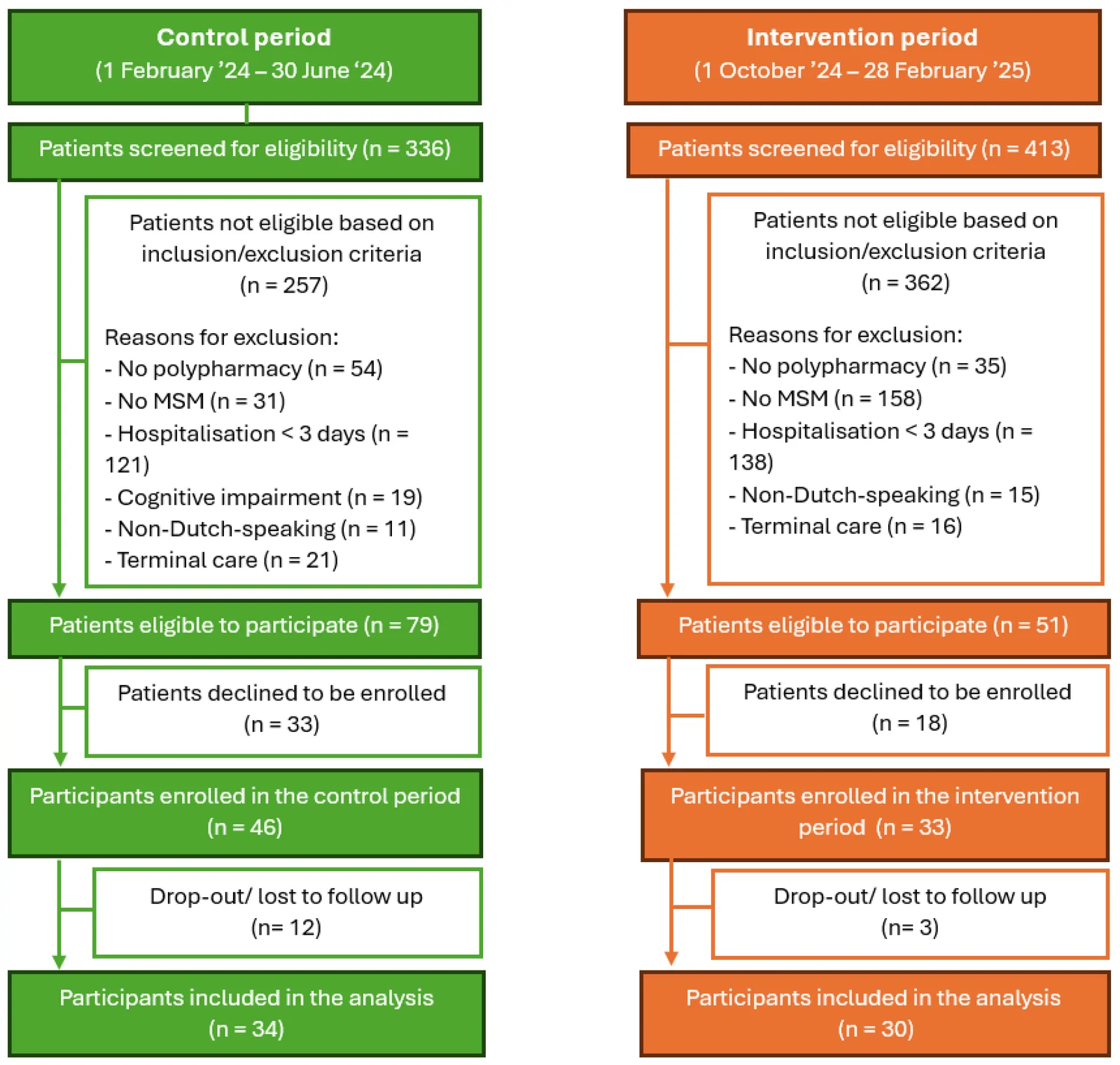
Flowchart of participants throughout the study.

Figure 2 illustrates the flow of participants throughout the study. During the control period, 336 patients were admitted, of whom 79 were eligible for inclusion and 46 consented to participate. Twelve patients were lost to follow-up, primarily due to disease progression, adverse effects, or death. During the intervention period, 413 patients were admitted, of whom 51 were eligible and 33 participated. Three patients dropped out due to difficulties with self-management, disease progression, and toxicity from pain medication, respectively. In total, 51 of the 130 eligible patients (39.2%) declined participation, most commonly due to lack of time or energy associated with intensive treatment. Patients who declined to participate, were significantly older (median 67 years [43–86] vs. median 64 years [22–84], *p* = 0.030). Dropouts used significantly more medicines than participants who completed the study (median 7 [5–16] vs. median 5 [5–18], *p* = 0.014). Loss to follow-up differed between study groups: 12 of 46 participants (26.1%) in the control group versus 3 of 33 (9.1%) in the intervention group.

Table 1 presents the socio-demographic characteristics of participants. No baseline differences were observed between control and intervention groups, except for the number of medications at hospital admission, which was lower in the intervention group (median 5 [5–10]) than in the control group (median 6 [5–18]). During hospitalisation, patients in the intervention group self-managed a median of 8 medications.

### 3.2. Primary Outcome: Medication Adherence Based on Pill Counts

**Figure 3:**
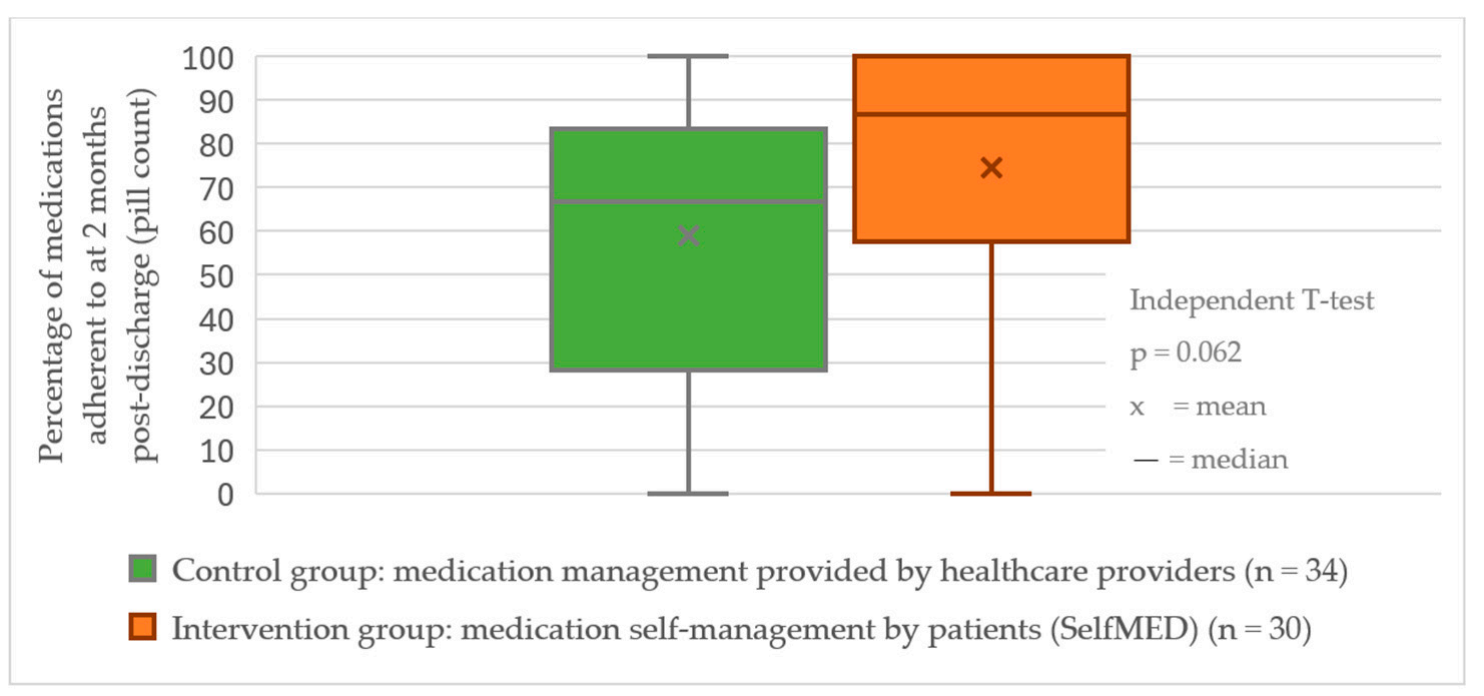
Boxplots of the percentage of medicines to which patients were adherent to (80–120% of doses taken).

Two months after discharge, a numerical difference was observed in the implementation phase of medication adherence, as measured by pill counts. Using a threshold of 80–120% of doses taken, patients in the control group were adherent to an average of 59.1% of their medications, compared with 74.5% in the intervention group (Figure 3). This difference was not statistically significant (*p* = 0.062). The mean difference was 15.4 percentage points (95% CI: −0.8 to 31.7); Cohen’s d = 0.48 (95% CI: −0.03 to 0.98). After adjustment for gender, age, educational level, and number of prescribed medications at admission, the estimated difference remained similar (B = 17.0, 95% CI −0.4 to 34.4, *p* = 0.055). No covariate was significantly associated with medication adherence (see Supplementary Table S1).

Sensitivity analyses examining the impact of missing outcome data showed that the estimated between-group difference (intervention−control) ranged from −2.0 to 24.1 percentage points, depending on the assumptions made regarding participants lost to follow-up. Only the worst-case scenario, in which all dropouts were assumed to be non-adherent, yielded a statistically significant difference (see Supplementary Table S2).

Medication adherence did not differ significantly between ATC level 1 categories (*p* = 0.075) (see Table 2). Nevertheless, patients were most frequently adherent to medicines in the category of antineoplastic and immunomodulating agents, followed by medicines for the respiratory system. In contrast, adherence was lowest for medicines acting on the nervous system, the musculoskeletal system and the alimentary tract and metabolism. When comparing intervention and control groups, no significant differences in adherence were observed across ATC categories after Bonferroni correction.

### 3.3. Secondary Outcomes

#### 3.3.1. Medication Adherence Based on Self-Report Methods

*Medication diary*

A significant difference in MSM problems reported in the diary was observed: 26.5% of participants in the control group reported problems, compared with only one participant (3.3%) in the intervention group (*p* = 0.015). The odds of reporting a problem were significantly lower in the intervention group compared with the control group (OR = 0.10, 95% CI 0.01 to 0.81). In the control group, the most reported problems were experiencing side effects, followed by forgetfulness and difficulties with administration. In the intervention group, forgetfulness was the only problem reported.

*ProMAS questionnaire*

Based on ProMAS questionnaire scores, no differences in medication adherence were observed between the control and intervention groups at baseline (median 13 [2–17] vs. median 13.5 [0–18], *p* = 0.961; Hodges–Lehmann median difference = 0; 95% CI −2 to 2) or at two months post-discharge (median 13 [9–18] vs. median 14 [0–18], *p* = 0.957; Hodges–Lehmann median difference = 0; 95% CI −1 to 2). Furthermore, no statistically significant within-group change in adherence was found between baseline and two months post-discharge (median 13 [0–18] vs. median 13 [0–18], *p* = 0.105; Hodges–Lehmann median difference = 0.5; 95% CI 0–1).

#### 3.3.2. Medication Knowledge

There were no differences in medication knowledge between the control and intervention groups at baseline (median 17 [9.5–19] vs. median 17 [15-20], *p* = 0.317; Hodges–Lehmann median difference = 0.5; 95% CI −1 to 0.5) or at two months post-discharge (median 17.5 [11.5–20] vs. median 18 [11.5–20], *p* = 0.837; Hodges–Lehmann median difference = 0; 95% CI −1 to 1). In addition, no statistically significant difference in knowledge was observed between baseline and two months post-discharge (median 17 [9.5–20] vs. median 17.5 [11.5–20], *p* = 0.158; Hodges–Lehmann median difference = 0.25; 95% CI 0 to 0.75).

#### 3.3.3. Medication Self-Management

The intervention group reported fewer MSM problems than the control group at two months post-discharge (mean 8.8 [SD 5.27] vs. mean 10.5 [SD 6.44]; mean difference 1.8; 95% CI −1.18 to 4.72), although the difference was not statistically significant (*p* = 0.235). Similarly, there was no statistically significant difference in MSM problems between baseline and two months post-discharge (mean 8.8 [SD 5.29] vs. mean 9.5 [SD 5.72], *p* = 0.260; 95% CI −2.05 to 0.56).

#### 3.3.4. Patient Satisfaction

Patients in the control group reported high satisfaction with medicines being stored, prepared, and administered by nurses during hospitalisation (median score 9/10 [5–10), while patients in the intervention group expressed high satisfaction with the opportunity to self-manage their medicines (median score 9/10 [4–10]; Hodges–Lehmann median difference 0; 95% CI 0 to 1). No significant difference in satisfaction was observed (*p* = 0.889). In addition, no significant differences were observed between groups in satisfaction with information received during hospitalisation (median 9/10 [3–10] vs. 9/10 [4–10], *p* = 0.525; Hodges–Lehmann median difference 0; 95% CI 0 to 1) or at discharge (median 9/10 [3–10] vs. 9/10 [5–10], *p* = 0.505; Hodges–Lehmann median difference 0; 95% CI 0 to 1). Similarly, satisfaction with support and guidance during hospitalisation did not differ between groups (median 9/10 [3–10] vs. 9/10 [5–10], *p* = 0.794; Hodges–Lehmann median difference 0; 95% CI 0 to 1). However, a significantly higher proportion of patients in the intervention group felt sufficiently prepared to self-manage their medicines at home compared with those in the control group (93.1% vs. 71.4%, *p* = 0.041; OR = 5.4; 95% CI 1.03 to 28.23).

#### 3.3.5. Healthcare Service Utilisation Two Months Post-Discharge

Table 3 shows no differences in healthcare service utilisation within two months post-discharge between intervention and control groups. Approximately 59% of patients were readmitted to hospital during this two-month period. Hospital readmissions were mainly due to oncological treatment (97%), not medication use or MSM problems. General practitioners were consulted in 67% of cases: in 50% for non-medication-related reasons and in 45% for prescription renewals. Visits to a community pharmacy were common (80%).

#### 3.3.6. Healthcare Providers’ Satisfaction and Workload

A total of 17 healthcare providers completed the questionnaire on satisfaction and workload (see Table 4 for socio-demographic characteristics).

Perceived feasibility and time investment of medication management activities did not differ significantly between intervention and control periods (see Table 5). Overall, 56.3% of healthcare providers expressed their preference towards MSM by patients, 31.3% preferred medication management by healthcare providers, and 12.4% reported no preference. Table 6 lists the advantages and disadvantages of both working methods as reported by healthcare providers.

## 4. Discussion

This study evaluated the effect of an in-hospital MSM intervention, SelfMED, on medication adherence and related outcomes at the patient-, healthcare provider-, and healthcare system levels. Although medication adherence rates were numerically higher in the intervention group than in the control group two months after hospital discharge, the difference was not statistically significant. Similarly, no statistically significant differences were observed between groups in medication knowledge, satisfaction, workload, or healthcare service utilisation. These findings are not fully consistent with previous studies, which reported positive effects of in-hospital MSM on medication adherence, knowledge, workload and satisfaction among patients who self-managed their medicines [19,20,23,41]. Randomised controlled trials in patients with chronic conditions and cardiovascular inpatients reported improved adherence after MSM programmes [19,20]. In contrast, reviews on in-hospital MSM reported mixed findings regarding medication adherence, with some studies demonstrating improvements while others found no statistically significant differences [42,43]. Improved medication knowledge has been reported in both oncology and non-oncology populations, but mostly when assessed at discharge [19,23,44]. Previous studies also reported high patient satisfaction with in-hospital MSM [22,45]. However, comparisons should be made cautiously, as prior studies often involved shorter follow-up periods, different patient populations, and varying intervention components.

The most important consideration when interpreting these findings is the small achieved sample size. Only 64 participants completed follow-up, approximately 14% of the 470 participants originally planned. The study was therefore substantially underpowered for its primary outcome, and the non-significant result does not provide evidence that SelfMED has no effect on medication adherence. Rather than statistical significance alone, the magnitude and precision of the estimate are informative: the observed difference of 15.4 percentage points was accompanied by a wide 95% confidence interval, which is compatible both with a negligible effect and with a substantial benefit of SelfMED. The present data can therefore neither confirm nor rule out a clinically relevant effect, and this uncertainty warrants further investigation in an adequately powered study.

Two further aspects should be considered when interpreting the primary outcome. First, because the control and intervention cohorts were recruited sequentially in different calendar periods, the design does not allow a potential intervention effect to be disentangled from temporal, organisational or clinical changes. Therefore, all between-group differences reported in this study should be interpreted in this light. Second, patients in the intervention group were prescribed significantly fewer medicines at admission than those in the control group. Because a higher number of prescribed medicines may increase medication regimen complexity, this imbalance could have favoured the intervention group. However, adherence was assessed based on the medication regimen prescribed at discharge, and the number of prescribed medicines at discharge was comparable between groups. In addition, the number of prescribed medicines was not associated with adherence in the regression analyses, and the adjusted between-group difference was similar to the unadjusted estimate. Nevertheless, given the non-randomised design, sequential recruitment and small sample, residual confounding by this baseline imbalance and by unmeasured factors cannot be excluded.

Furthermore, in this study, no statistically significant differences in perceived feasibility and time investment were observed between MSM during hospitalisation and healthcare provider-led medication management. Earlier research showed that while initiating MSM can be time-consuming, time savings occur during medication dispensing, administration, and discharge preparation, as counselling is integrated throughout the hospital stay [46]. Other studies have shown reductions in nurses’ time spent on medication administration [19,41]. However, these studies typically allowed patients to use their own home medication during hospitalisation. As Belgian legislation stipulates that all medication for hospitalised patients must be supplied by the hospital pharmacy [47], healthcare providers need additional time to supply medication to patients in a way that simulates the home situation. Allowing patients to use their own medication during hospitalisation could enhance feasibility and reduce time burden.

More patients in the intervention group reported feeling adequately prepared to self-manage their medication after discharge. Improved patient preparedness could represent one mechanism through which in-hospital MSM might contribute to continuity of care and patient empowerment beyond the hospital setting. However, this finding should be interpreted with caution. It was based on a single self-reported item and the estimate was imprecise (OR = 5.4, 95% CI 1.03 to 28.23) with a lower confidence limit only close to the null value. Furthermore, we acknowledge that the greater preparedness reported by patients may partly reflect increased attention from healthcare professionals during the training and implementation period (Hawthorne effect) rather than being attributable solely to the specific effects of SelfMED. Nevertheless, supporting patients to develop self-management competencies during hospitalisation could represent an important step towards safer transitions of care.

### 4.1. Strengths and Limitations

To our knowledge, this is the first study to evaluate the effect of the SelfMED intervention on medication adherence following hospital discharge. A major strength of this study is its focus on a high-risk population of hospitalised patients with polypharmacy.

To minimise the risk of contamination bias, a pre–post design was employed. Because participants were recruited sequentially rather than in parallel, the control and intervention groups were exposed to different calendar periods, which may have introduced temporal confounding related to seasonal variation in patient case-mix, changes in oncology treatment protocols or increasing staff experience and familiarity with study procedures over time. However, both study phases were conducted using identical eligibility criteria, outcome measures, and data collection procedures. In addition, the absence of randomisation and blinding may have increased the risk of confounding due to baseline differences between groups. Alternative designs that preserve randomisation while limiting contamination, such as cluster-randomised or stepped-wedge designs, would not have been feasible within a single ward. Nevertheless, the chosen design reflects real-world clinical practice more accurately.

Despite screening nearly 750 patients, the target sample size was not reached, resulting in insufficient statistical power. The achieved sample size of 64 participants corresponds to only approximately 14% of the originally planned total sample (470), which substantially increases the risk of Type II error. The main reason for under-recruitment was the high proportion of patients who did not meet the strict inclusion and exclusion criteria, primarily due to short lengths of stay. The study aimed to include patients with complex medication regimens who typically experience longer hospitalisations, providing more opportunities to fully support patients in developing MSM competencies.

While a multicentre study could have increased sample size, it would have required additional resources for coordination across sites, monitoring and follow-up. A single-centre approach ensured consistent implementation of the intervention, reliable data collection, and rigorous control of study procedures within the available resources (i.e., funding and personnel).

Differential attrition between groups may have introduced selection bias. Participants lost to follow-up were taking significantly more medications than those who completed the study, and loss to follow-up was more frequent in the control group than in the intervention group. If a higher number of medications is associated with lower adherence, the disproportionate loss of participants with more complex medication regimens from the control group may have resulted in a control sample that was relatively more adherent than the original study population. Consequently, this attrition pattern would be expected to attenuate, rather than explain, the observed between-group difference in adherence. However, the potential impact of this differential attrition on the study findings cannot be excluded and should be considered when interpreting the results. The sensitivity analyses further demonstrated that the estimated between-group difference was sensitive to assumptions regarding participants lost to follow-up, reinforcing the exploratory nature of the findings.

The study population was limited to oncology patients with polypharmacy who were considered eligible for medication self-management according to the SelfMED assessment. Therefore, the findings may not be generalisable to patients with substantial cognitive or physical limitations or those who rely on formal or informal caregivers for medication management.

SelfMED’s components (i.e., assessment, monitoring system, and supportive measures) were paper-based, which may have influenced healthcare providers’ perceptions of administrative burden and feasibility. Digitising these components and integrating them into the hospital’s electronic health record could streamline workflows. This would not only facilitate data sharing among healthcare providers but also enable seamless exchange between patients’ medical records and the SelfMED tools, thereby reducing administrative burden, saving time, and improving feasibility for healthcare professionals. However, evidence of the intervention’s effectiveness is needed to justify the financial investment required for such digitalisation.

Medication adherence was primarily assessed through pill counts, applying a threshold of 80% pills taken to classify patients as adherent or non-adherent. However, the appropriateness of this threshold may vary across medication types and classes [38,39]. Ideally, adherence thresholds should be evidence-based and tailored to specific medications, informed by studies linking adherence levels to relevant clinical outcomes. In the absence of such data for all medications, a pragmatic and somewhat arbitrary threshold was applied.

The analyses by ATC category were exploratory. Although a Bonferroni correction was applied to account for multiple comparisons, observations were not fully independent, as multiple medications could originate from the same participant, and the limited sample size precluded clustered analytical approaches such as mixed-effects or generalised estimating equation models.

As multiple secondary and exploratory analyses were performed without adjustment for multiple comparisons, there is an increased risk of Type I error. Therefore, statistically significant findings from these analyses should be interpreted with caution.

Furthermore, the statistical analyses were not performed blinded to group allocation, which represents a potential source of detection bias.

Most secondary outcomes were evaluated using self-reported data, introducing a potential risk of social desirability bias. Despite their limitations, self-report instruments remain valuable for capturing patient experiences and perspectives, which are essential for evaluating patient-centred interventions.

As SelfMED is a multi-component intervention, the present study cannot disentangle the individual effects of self-management practice, patient education, and healthcare professional support on the observed outcomes.

### 4.2. Recommendations for Further Research

Multicentre studies with larger samples are needed to confirm these findings. Future work could assess implementation outcomes, such as acceptability and appropriateness. Additionally, the implementation process itself, including barriers and facilitators, could be examined using frameworks such as the Consolidated Framework for Implementation Research (CFIR). Expanding SelfMED to patients with shorter hospital stays and less complex medication regimens may enhance scalability and sustainability in clinical nursing practice.

### 4.3. Implications for Policy and Practice

Currently, MSM is not systematically facilitated within Belgian hospitals. Patients are rarely supported during hospitalisation in developing the skills and confidence needed to take responsibility for medication management after discharge. In this study, patients in the intervention group more often reported feeling prepared to self-manage their medicines at home. Considering this finding, along with the comparable feasibility and workload between the two approaches and the fact that more than half of healthcare providers expressed a preference for MSM, structured in-hospital MSM support may represent a promising direction for hospital practice. However, given the exploratory nature and limited statistical power of this study, adequately powered multicentre studies are needed before SelfMED can be recommended as a standard component of care.

## 5. Conclusions

This exploratory, single-centre, non-randomised study did not demonstrate a statistically significant effect of the SelfMED intervention on its primary outcome, medication adherence, nor on medication knowledge, patient or staff satisfaction, workload, or healthcare service utilisation. The numerically higher, but not statistically significant, adherence in the intervention group, together with fewer reported MSM problems and greater patient preparedness to self-manage medication at home, suggests possible benefits of SelfMED that warrant further investigation. However, because the achieved sample size was substantially smaller than planned and the study was underpowered for the primary outcome, these findings should be regarded as exploratory rather than confirmatory. Adequately powered multicentre randomised or pragmatic implementation studies, in more diverse patient populations, are needed before firm conclusions can be drawn regarding SelfMED’s effectiveness and before its integration into standard hospital practice can be recommended.

## Figures and Tables

**Table 1:** Participant characteristics.

Characteristics	Total (n = 64)	Control Group (n = 34)	Intervention Group (n = 30)	*p*-Value
**Age**, mean (SD)	60.1 (12.1)	59.9 (12.2)	60.3 (12.3)	0.883 ^b^
**Gender (female)**, n (%)	42 (65.6)	23 (67.6)	19 (63.3)	0.795 ^c^
**Highest educational level (EQF** ^a^**)**, n (%)				0.816 ^d^
No education	5 (7.8)	2 (5.9)	3 (10.0)	
EQF level 1	2 (3.1)	1 (2.9)	1 (3.3)	
EQF level 2/3/4	26 (40.6)	16 (47.1)	10 (33.3)	
EQF level 5/6	24 (37.5)	12 (35.3)	12 (40.0)	
EQF level 7	7 (10.9)	3 (8.8)	4 (13.3)	
**Hospitalisation type**, n (%)				1.000 ^c^
Planned	21 (32.8)	11 (32.4)	10 (33.3)	
Unexpected	43 (67.2)	23 (67.6)	20 (66.7)	
**Reason for admission,** n (%)				0.231 ^d^
Treatment	36 (56.3)	19 (55.9)	17 (56.7)	
Examination	11 (17.2)	8 (23.5)	3 (10.0)	
Medication review	6 (9.4)	4 (11.8)	2 (6.7)	
Observation	9 (14.1)	2 (5.9)	7 (23.3)	
Other	2 (3.1)	1 (2.9)	1 (3.3)	
**Number of prescribed medicines on admission,** median [range]	5 [5–18]	6 [5–18]	5 [5–10]	0.010 ^e^
**Number of prescribed medicines at discharge,** median [range]	8 [5–21]	8 [5–19]	9 [5–21]	0.541 ^e^
**Number of self-managed medicines during hospitalisation,** median [range]	/	/	8 [5–11]	/

^a^ European Qualifications Framework; Level 1: primary education, Level 2: lower secondary education, Level 3: upper secondary vocational education, Level 4: upper secondary education, Level 5: associate’s degree, Level 6: bachelor’s degree, Level 7: master’s degree, ^b^ Independent *T*-test, ^c^ Fisher’s Exact test, ^d^ Pearson Chi-square test, ^e^ Mann–Whitney U test.

**Table 2:** Percentage of medicines to which patients were adherent, classified by ATC first level.

ATC 1st Level		Number of Prescribed Medicines	% of Medicines Adherent to (80–120% Doses Taken)	*p*-Value
A	Alimentary tract and metabolism	101	60.4%	0.075 *
B	Blood and blood forming organs	13	69.2%	
C	Cardiovascular system	76	71.1%	
G	Genito urinary system and sex hormones	14	78.6%	
H	Systemic hormonal preparations	39	74.4%	
J	Anti-infectives for systemic use	25	68.0%	
L	Antineoplastic and immunomodulating agents	17	94.1%	
M	Musculoskeletal system	10	60.0%	
N	Nervous system	82	56.1%	
R	Respiratory system	11	81.8%	

* Chi-squared test. Note: This table represents an unadjusted, exploratory descriptive analysis without correction for intra-patient clustering. Data are pooled from both study groups.

**Table 3:** Healthcare service utilisation within two months post-discharge.

	Control Group (n = 34)	Intervention Group (n = 30)	Median Difference ^b^	95% CI	*p*-Value
**Number of hospital readmissions**, median [range]	1 [0–6]	1 [0–5]	0	−1 to 0	0.292 ^a^
**Consultations with general** **practitioner**, median [range]	2 [0–7]	2 [0–4]	0	−1 to 1	0.877 ^a^
**Pharmacist visits**, median [range]	2 [0–18]	2 [0–5]	0	−1 to 1	0.774 ^a^

^a^ Mann–Whitney U Test; ^b^ Hodges–Lehmann median difference.

**Table 4:** Characteristics of healthcare providers.

Variables	n (%)
**Healthcare provider type** (n = 17)	
Nurse	10 (58.8)
Physician	7 (41.2)
**Gender (female)** (n = 17)	12 (70.6)
**Age** (n = 17)	
20–30 years	6 (35.3)
31–40 years	4 (23.5)
41–50 years	3 (17.6)
51–60 years	3 (17.6)
>60 years	1 (5.9)
**Work experience** (n = 13) *	
0–5 years	6 (46.2)
6–10 years	2 (15.4)
11–20 years	3 (23.1)
21–30 years	1 (7.7)
>30 years	1 (7.7)

* Denominators may vary because of missing responses.

**Table 5:** Mean scores (out of 10) for feasibility and time investment of medication management activities during control and intervention periods (as perceived by nurses).

		Medication Management by Healthcare Providers ^a^	Medication Self- Management by Patients ^a^	Mean Difference (HCP−Patient) ^b^	95% CI	*p*-Value ^c^
**Completing assessment upon admission (n = 9)**	Feasibility	/	7.1 [1.64]	/	/	/
	Time investment	/	5.8 [2.92]	/	/	/
**Ordering medication from hospital pharmacy (n = 9)**	Feasibility	8.6 [1.06]	8.0 [1.93]	+0.6	−0.4 to 1.6	0.180
	Time investment	4.9 [3.14]	5.8 [2.87]	−0.9	−3.7 to 1.9	0.480
**Dispensing and/or preparing medication (n = 9)**	Feasibility	8.8 [0.71]	7.9 [0.99]	+0.9	−0.1 to 1.8	0.064
	Time investment	4.8 [2.87]	5.8 [2.55]	−1.0	−4.6 to 2.6	0.536
**Monitoring medication** **intake (n = 9)**	Feasibility	7.9 [2.10]	7.5 [1.31]	+0.4	−0.9 to 1.6	0.504
	Time investment	5.0 [1.93]	5.3 [1.98]	−0.3	−1.6 to 1.1	0.685
**Providing information and education (n = 9)**	Feasibility	8.4 [1.13]	8.3 [1.12]	+0.1	−0.1 to 0.4	0.347
	Time investment	5.6 [1.59]	6.1 [1.76]	−0.5	−1.6 to 0.5	0.276

^a^ Values are presented as mean [standard deviation]. ^b^ Mean difference between healthcare-provider-led and patient self-managed scores; positive values indicate higher scores for healthcare-provider-led management ^c^ *p*-values were obtained using paired-samples *t*-tests. Note: Denominators represent the number of nurse respondents with complete data for the respective paired comparison, as the activities presented in this table were primarily performed by nurses.

**Table 6:** Healthcare providers’ perceived advantages and disadvantages of usual care versus SelfMED intervention.

	Advantages	Disadvantages
Medication management by healthcare providers (usual care)	+ More control and overview of medication intake for nurses + Increased safety: reduced risk of errors, more suitable for patients with cognitive impairment or vulnerable health status + Accuracy and consistency ensure correct timing and dosing + Clear communication and patient education about medication	− Delayed medication administration − Less patient autonomy − Less patient involvement − Time-consuming for nurses (i.e., administration) − Less patient insight into and knowledge of medication
Medication self-management by patients (SelfMED)	+ Promotes patient autonomy and independence + Enhances patient involvement + Provides opportunities for patients to learn about their medication + Time-saving for nurses + Timely intake by patient + Increased self-control for patients	− Reduced control over medication intake by nurses − Lack of medication overview by nurses − Less suitable for patients with limited medication knowledge − Risk of errors by patients

## Data Availability

Data are available from the authors upon reasonable request due to privacy and ethical restrictions. Access to participant-level data was granted solely for the purposes of this study under the conditions set by the ethics committee.

## Supplementary Materials

The following supporting information can be downloaded at https://www.mdpi.com/article/10.3390/healthcare14203436/s1, Supplementary Table S1: Linear Regression; Supplementary Table S2: Sensitivity Analysis.

## Abbreviation

The following abbreviations are used in this manuscript:

MSM	Medication self-management
