# Supplementary material for: The Effect of Medication Self-Management During Hospitalisation (SelfMED) on Medication Adherence Post-Discharge in Oncology Patients with Polypharmacy: A Prospective, Exploratory, Single-Centre, Non-Randomised Pre–Post Intervention Study with Sequential Control and Intervention Cohorts
Source: Healthcare (Basel). 2026 Oct 14;14(20):3436. doi: 10.3390/healthcare14203436 (PMC13704667; doi:10.3390/healthcare14203436)
Supplement: Supplementary file 1 [file healthcare-14-03436-s001.zip › Supplementary Table S1 - Linear regression.pdf]

Supplementary Table 1: Factors associated with medication adherence: univariate and multiple linear regression analyses

Variables	Descriptive analysis		Univariate linear regression			Multiple linear regression (n= 64, R^2= 0.116, Adjusted R^2 = 0.040, p =0.198)				
	Percentage of medication adherence	Independent t test p-value	B	Bèta	p-value	B	Bèta	CI 95% B (lower)	CI 95% B (upper)	p-value
**Study group**		0.062	15.4	0.234	0.062	17.0	0.258	-0.355	34.404	0.055
Intervention (n = 30)	74.5									
Control (n = 34) - ref	59.1									
**Gender**		0.101	14.3	0.207	0.101	15.0	0.217	-2.547	32.612	0.092
Male (n = 22)	75.7									
Female (n = 42) - ref.	61.4									
**Education**		0.627	4.2	0.062	0.627	1.9	0.028	-15.756	19.573	0.830
≥ than EQF level 5 (n = 23)	69.0									
< than EQF level 5 (n = 41) – ref.	64.8									
	Pearson correlation	P-value								
**Age** (n = 64)	-0.036	0.778	-0.098	-0.036	0.778	-0.24	-0.088	-0.941	0.460	0.495
**Number of prescribed medicines on admission** (n = 64)	0.044	0.732	0.612	0.044	0.732	1.745	0.124	-1.934	5.423	0.346
