# Supplementary material for: The Effect of Medication Self-Management During Hospitalisation (SelfMED) on Medication Adherence Post-Discharge in Oncology Patients with Polypharmacy: A Prospective, Exploratory, Single-Centre, Non-Randomised Pre–Post Intervention Study with Sequential Control and Intervention Cohorts
Source: Healthcare (Basel). 2026 Oct 14;14(20):3436. doi: 10.3390/healthcare14203436 (PMC13704667; doi:10.3390/healthcare14203436)
Supplement: Supplementary file 1 [file healthcare-14-03436-s001.zip › Supplementary Table S2 - Sensitivity analysis.pdf]

Supplementary Table 2: Sensitivity analyses for the primary outcome: medication adherence two months post-discharge.

Scenario	Control group	Intervention group	Difference intervention - control (95% CI)	p-value*
Complete-case analysis (main analysis)	(n = 34)<br>59.1%	(n = 30)<br>74.5%	15.4 (−0.8 to 31.7)	0.062
A. Worst case (all dropouts 0% adherence)	(n = 46)<br>43.7%	(n = 33)<br>67.8%	24.1 (6.8 to 41.4)	0.007
B. Worst case for the hypothesis (control dropouts 100%, intervention dropouts 0% adherent)	(n = 46)<br>69.8%	(n = 33)<br>67.8%	−2.0 (−18.0 to 14.0)	0.803
C. Cross-over (control dropouts 74.5%, intervention dropouts 59.1%, i.e., the observed mean of the other group)	(n = 46)<br>63.1%	(n = 33)<br>73.1%	10.0 (−3.5 to 23.5)	0.144

* Independent-samples-t-test

Note: Values are mean percentages of prescribed chronic medications for which participants were classified as adherent (i.e., 80–120% of prescribed doses taken). Scenarios A–C include all enrolled participants, with imputed values for participants lost to follow-up (control n = 12; intervention n = 3).
